# Nurses’ perceptions on the effects of high nursing workload on patient care in an intensive care unit of a referral hospital in Malawi: a qualitative study

**DOI:** 10.1186/s12912-022-00918-x

**Published:** 2022-06-01

**Authors:** Zione Banda, Mirriam Simbota, Chimwemwe Mula

**Affiliations:** University of Malawi, Kamuzu College of Nursing, Blantyre, Malawi

**Keywords:** Nursing workload, Patient safety, Quality of care

## Abstract

**Background:**

The Malawi health system has taken numerous actions to reduce high nurse workloads, despite this, shortage of nurses especially in critical care settings still persists due to lack of prioritisation of critical care. Therefore, it is important to understand the effects of high nursing workload in Intensive Care Unit (ICU). This study aimed at exploring the perceptions of nurses regarding the effects of high nursing workload on patient care in ICU at Queen Elizabeth Central Hospital.

**Methods:**

This qualitative descriptive study was conducted in a general ICU at Queen Elizabeth Central Hospital in Blantyre, Malawi. A purposive sample of 12 nurses working in the ICU was selected. Participants included full-time nurses working in the ICU. A total of 10 In-depth interviews were conducted to collect data upon which data saturation was reached. A semi-structured interview guide was used for data collection. Data was analysed manually using thematic analysis method by Braun & Clarke.

**Results:**

Study findings indicated that high nursing workload compromises the delivery of quality nursing care to critically ill patients, compromises patient safety and has negative impact on nurses’ wellbeing.

**Conclusion:**

The study findings portray that nurses are aware of the negative effects that high nursing workload has on patient care. The study findings support the need for more ICU nurses in order to reduce nurse workloads and the need for nurse managers and policy makers to develop strategies to manage nurse workloads and its effects on patient care.

## Introduction

Intensive care units (ICUs) require higher levels of nurse staffing than other health care environments, because of the demands of care needed for critically ill patients [1]. The ICU is an environment that provides care for patients with severe clinical conditions that require ventilation and acute medical clinical care [2]. Evidently, issues of high nursing workloads are more definite in ICUs compared to other areas in hospitals due to the extensive and intense duties that ICU nurses have both physically and mentally [3]. Increased patient acuity and complexity of illness requires constant surveillance as well as medication treatments that are always accompanied by increased physiological and psychological needs that demand comprehensive and holistic nursing care [3–5]. ICU patients need to receive special care such as ventilation, drug administration etc. for a long time, which highlights the huge role of ICU nurses [6]. Apart from direct patient care; ICU nurses are involved in a variation of other tasks including making pharmacy orders, filling of updates of blood sample results, reception of families of newly admitted patients and attending to time consuming calls of family members. These administrative roles also increases the amount of workload ICU nurses are exposed to [7].

There is a link between nursing workloads and quality of care in critical care areas where it has been shown that high nursing workloads negatively affect both patient care and nurse outcomes [8–10]. Reports of missed care, care left undone and poor documentation of care have been made [11, 12]. Studies have also shown that high nursing workload is significantly associated with increased incidents of falls, medication errors, unplanned extubations [13–15], increased acquisition of infections [16], decreased odds of survival and affects patient safety in general [17, 18]. Emerging research has also associated improved nurse-patient ratios and reduced nursing workloads with improved patient outcomes [19].

While the Malawi health sector has made tremendous progress in improving patient outcomes, the shortage of staff persists to be a major challenge [20]. With a 45% vacancy rate across the public health sector, vacancy rates for Registered Nurses and Nurse Midwife Technicians being 66% and 60% respectively, the government is still striving to increase the supply of nurses in order to provide quality health services in line with best global practices [20]. In the situation of shortage of nurses, not many nurses are allocated to work in critical care areas, which might be attributed to the lack of prioritisation or lack of interest in critical care [21].Even though evidence has shown that high nursing workload is associated with unfavourable outcomes, limited research has been published in Malawi on the effects of high nursing workload on patient care. Understanding the effects of nursing workload in the ICU, especially from nurses’ perspectives, is an urgent undertaking, given nurse shortages and the numerous associations between workload, quality of care and patient safety. Understanding of such effects will help nurses and nurse managers to recognise and be able to address the issues related to high nursing workload in the ICU and improve the provision of quality and safe care. This study aimed at exploring and describing nurses’ perceptions of the effects of high nursing workload in ICU in Malawi.

## Methods

### Study design

This study employed a descriptive qualitative design as described by Polit and Beck [22]. ICU nurses are ‘insiders’ in as far as Intensive Care is concerned and their perceptions provide an insider view, as such a qualitative approach examined in detail the nurses’ perceptions on nursing workload and quality of care from those who experience the high workload, this helped the researchers to gain insight and find results that will guide practice. The study was documented by using a recommended reporting system; Consolidated criteria for reporting qualitative research (COREQ) [23].

### Study setting

The study was conducted at Queen Elizabeth Central Hospital (Q.E.C.H.) which is situated in Blantyre city in the southern region of Malawi. Q.E.C.H is a referral hospital with a bed capacity of 1,200 and serves a population of approximately 5.5 million [24]. At the time of data collection; The hospital had 1 adult ICU which had 4 beds and 3 stepdown high dependency units for adults which had 4 beds each. On average, the ICU was admitting 350 patients annually with a 1:2 nurse patient ratio.

### Study participants and sampling

Inclusion criteria for this study included: working in the ICU at Q.E.C.H. for more than three months and willingness to participate in the study by providing a signed consent. Excluded from participating in the study were temporary nurses working on month-to-month basis and those working on locum basis (substitute nurses). These nurses were excluded as they do not work full time in the ICU and may not have entirely experienced the phenomenon under study. For sampling, study participants were purposively selected, the researcher contacted the ICU under study and used face-to-face approach to inform 12 nurses of the study and the participant inclusion criteria. All 12 participants that were approached met the study’s inclusion criteria and responded to the researcher that they were interested. Qualitative research is labour intensive and large samples are time consuming and impractical, a sample for a rigorous qualitative study as such, is not very large [25]. Data saturation was reached upon interviewing 10 nurses.

### Ethical consideration

Informed consent was obtained from all study participants when conducting this study. We observed participant confidentiality by anonymising our data, participants were given codes for identification (e.g., participant 1 NMT; for Nurse midwife technician or RN for Registered Nurse). The study was approved by College of Medicine Research and Ethical Committee (COMREC) reference number P.05/19/269. We confirm that all study methods were performed in accordance with the relevant guidelines and regulations.

### Data collection

Data was collected for a period of four weeks from 19^th^ July, 2019 to 9 August, 2019 by the first author; ZB through in-depth interviews, which were recorded using a digital audio recorder, and where necessary, some of the information was handwritten in order to collect adequate data as much as possible. The researcher conducted the interviews with the aid of a semi-structured interview guide. The interview guide questions were formulated from concepts from literature with an aim of addressing perceptions on the effects of high nursing workload on patient care in the ICU. Unplanned and unanticipated probes were used as supplementary questions. Unplanned probes arose from the context of the interview. Interviews were conducted in the resource room in the ICU, only the researcher and the participant were present during interviews and each interview lasted 40–45 min. Pre-testing of the interview guide was done at Zomba Central Hospital (ZCH) using three nurses working in the ICU. The hospital was chosen as it is also a referral hospital. During interviews; the researcher explained the purpose of the study to each and every participant before the interview and asked them to sign an informed consent form. The researcher then followed the semi structured interview guide. Questions included: *Describe how the current high workload influences your performance? How does it affect your performance? What are the effects/consequences on the provision of critical care? How does the high nursing workload you experience affect the safety of patients in the unit? What errors occur when providing care in regards to high nursing workload? What are the occurrences of missed care in regards to high nursing workload?* The researcher conducted one interview with one participant at a time and interviews were not repeated. The researcher kept a reflective journal with recorded field notes throughout the study. Participants were followed up for member checking purposes. The study results were shared to three participants in order to explore the credibility of the results. Participants checked for accuracy and resonance with their perceptions in the interpretations of the collected data and confirmed that it was is a true reflection of the information.

### Data analysis

Data was analysed manually according to Braun & Clarke’s thematic analysis [26]. Data analysis progressed following a step by step approach, Interviews were transcribed verbatim from a digital voice recorder within 24 h of data collection [27]. The transcripts were read and re-read in order to make sense of data while looking for patterns and meaning within the context of significant words or phrases, the researcher noted related phrases and units on participants’ perception of high workload; participants viewed high workload as negative. The next step involved generating initial codes which helped in organising the data in a meaningful and systematic way. Initial codes included; unplanned extubations, cardiac arrest, near fall situations, errors, compromised patient safety etc. Coding of data is essential in moving methodically to a slightly higher conceptual level [28]. This higher conceptual level enabled the researcher later to sort the items from different records in different ways, such as into similar and dissimilar groups and related features of the groups could be examined to gain insight into them [28].

The initial codes and all the relevant coded data extracts were sorted, collated and analysed. The different codes were clearly examined and recurring views were then combined and the researcher started to form overarching themes from the data. Poor quality care, compromised patient safety, unplanned extubations, cardiac arrests, impact on the nurse were identified as some of the themes. These themes were characterised by significance and predominantly described patterns in the data relevant to the research question. The researcher then reviewed the devised set of themes to ensure that they made sense and that themes were working in the context of the entire data set. Themes of unplanned extubations, cardiac arrests, near fall situations overlapped and all fell into the theme of compromised patient safety which was earlier identified. Themes of poor-quality care and leaving care undone were collapsed into a theme of “care compromised”. Themes were then defined and further refined with an aim of identifying the essence of the meaning of each theme.

## Results

Ten interviews with participants were conducted in order to explore their perceptions, upon which data saturation was reached. Presented below are the findings.

### General demographic data

We interviewed a total of 10 nurses working in the ICU. The ages of participants ranged from 30 to 43. Nurses had a working experience ranging from 3 to 8 years of working in the ICU. The majority [8] of the participants had a Diploma in Nursing and Midwifery, working as Nurse Midwife Technicians (NMTs) and a few [2] had a Bachelor’s Degree in Nursing midwifery, working as Registered Nurses (RNs). None of the participants interviewed had a qualification in critical care nursing.

Four themes emerged from the thematic analysis: Care compromised, compromised patient safety, impact on nurse capacity and impact on nurses’ wellbeing. Their description and supporting quotes are reflected in Table 1.

**Table 1:** Showing study results

Themes	Findings	Supporting Quotes
Care compromised	Nurses are unable to provide maximum quality care and leave care activities not considered priority undone	“We only prioritize some things; leaving the other things not done. Its better I do this and that…You can’t turn the patient because you have to run and check the other patient, it means you have left out the turning which is very important. And you can’t feed in time because you have to check if Airway Breathing and Circulation is okay on the other bed. I can abandon things like turning, feeding, mouth care, changing patients’ soiled linen; Because those are the things that I can do later” (Participant 6, NMT)
Compromised patient safety	Nurses perceived that the safety of ICU patients is compromised due to the high nursing workload experienced Nurses at times prepare the wrong medication when they are tired and exhausted	“At some point they are not safe, frankly speaking they are not safe…a patient had a cardiac arrest, we discovered that it was because of a delay in suctioning and that the tube had blocked. Had it been that we did the suctioning at the right time. this patient couldn’t have arrested, but because of the understaffing we were busy taking care of the other patient not knowing that the tube on the next patient was blocking. It was bad to us, because our intention is to save lives” (Participant 4, NMT) “My colleague was very tired and it was during the night, he wanted to prepare 10% dextrose, so he went to take the vails but he didn’t read on the vials. So instead of taking 50% dextrose he took lignocaine, I asked him “what are you doing?” and he said “am preparing 10% dextrose” then I said “look this is lignocaine”, he said “oh I didn’t notice that this is lignocaine” so he had to discard that and start all over again, later he said “what if you were not around, that means I could have killed the patient” but he said “it’s because am tired” (Participant 4, NMT)
Impact on nurse capacity	Perceptions indicated that high nursing workload affects nurses’ capacity to perform negatively	“We are always exhausted and the care which we give to the patient, I can confirm that it can’t be 100% correct” (Participant 9, NMT) “Performance is poor. The performance is poor, not because am poor as such, but it’s difficult because there is one hand where there should be 2 hands” (Participant 2, NMT)
Impact on nurses’ wellbeing	High nursing workload has both physical and psychological effects Physical impact—ICU patients attract a lot of nurses’ attention Psychological impact -Nurses perceived high nursing workload usually brings an increase in psychological issues; stress feelings of guilt, anger and discouragement	“High workload is not good. you have body pains, the legs are aching and you still have to run to help this one and help that one” (Participant 2, NMT) “It’s very stressful. Its stressful and also you tend to miss some other things like you give medication and you do not document because you were busy with something else” (Participant 1, NMT) At the end of the day, you become so emotional and you blame yourself sometimes you feel like being a nurse is a not good thing, because when you have a lot of work and when patients get negative outcomes, you feel bad about it so it affects us, it affects me negatively” (Participant 7, NMT)

## Discussion

Study findings in this paper reveal the effects of high nursing workload in ICU and are presented under the following themes; “care compromised”, “compromised patient safety”, “impact on nurse capacity” and “Impact on nurses’ wellbeing”.

The study findings depict that quality care is not achieved due to high workload which is in line with other literature [8]. Nurses were of the opinion that nursing procedures are mostly conducted in a rush in order to get to the next activity or to help the next patient which compromises both quality of care and the safety of patients resulting in Incidences of unplanned extubations. Unplanned extubation in ICU is a widely cited example of a potentially catastrophic and costly adverse event that is associated with increased morbidity and mortality [29]. In terms of patient injuries, the study found that nurses have never experienced a patient fall, when they notice a patient is about to fall, they rush to the bed. However, near fall situations were cited to be numerous. Carlesi et al., in a quantitative, analytical, cross-sectional study supports the association of high nursing workload with patient falls [14]. Patient falls, result in physical and psychological consequences and increases patients’ length of stay in ICU [30].

We also found that high nursing workload compromises care as nurses perceived they leave out care activities such as; patient feeding, patient turning, mouth care and linen changing when they have other activities to do. Studies have reported similar nursing activities to be left undone (or ‘missed’) when ICU nurse workloads are higher due to insufficient numbers of nurses in a shift which results in unfavorable patient outcomes including pressure ulcers, urinary tract infections, patient falls, critical incidents, poor quality of care and patient readmissions to ICU [12, 31].

The study findings reveal that patients develop or are at risk of developing Hospital Acquired Infections (HAIs) resulting from bed sores as nurses do not turn patients’ position in good time owing to the high workload. Furthermore, aseptic techniques are not properly followed as nurses usually have a lot of activities to perform with limited time. There is evidence that high nursing workload affects standard precautions for instance hand hygiene even in highly trained and well-staffed settings [32], confirming that the higher the nursing workload, the lower the compliance to standard precautions. Acquisition of HAIs in ICUs predisposes patients to higher mortality rates, additional adverse events, increases their length of stay in ICU and increases costs on health facilities, families and the society especially in sub-Saharan Africa where the burden of HAIs is already a great challenge [33, 34]. Considering the high demand on the few available ICU beds in Malawi, it is important to address and correct any relevant issues that increase patients’ length of stay in the ICU, including high nursing workload, to maximize the provision of intensive care to patients.

Our findings demonstrate that medication errors occur in the unit as a result of a high nursing workload. Work demands causes nurses to postpone medication administration, forget administering medication or preparing the wrong medication for patients when they are exhausted. Although it is argued that it is difficult to detect medical errors in ICUs as deteriorating patients’ health can be attributed to the patient’s underlying medical condition as opposed to nurses’ error, studies have attested the existence of a direct relationship between exhaustion and fatigue resulting from heavy workloads and the occurrence of errors among critical care nurses [35, 36]. It is claimed that medication errors may be more as a result of high-level task, as tasks demand the need to concentrate or multitask. Medication errors might be as a result of mediated interruptions, divided attention, and rushing which occurs when nurses experience a high-level task. Thus, a cognitive performance mechanism which is a component of nursing workload could be posited to explain medication errors [37].

Nurses in the present study perceived that the workload they experience is stressful, reduces their energy and strength which affects their performance in physical and mental care activities in the long run nurses further reported of fatigue and exhaustion which reduces their focus and level of concentration when providing care. Individual’s well-being and curtailed work performance may occur when there is high workload resulting in behavior changes such as reduced work pace [38]. Evidence has shown that chronically fatigued nurses experience changes in their alertness and concentration while providing patient care [39]. Efforts to decrease workplace stress of ICU nurses by focusing on facilitating peer collaboration, improving resource availability, and staffing ratios are likely to reduce stress levels.

High nursing workload was found not to only affect nurses’ performance; nurses further perceived that high nursing workload affects their wellbeing physically and psychologically. In regards to physical impact, nurses reported musculoskeletal problems including lower back pain, neck pain and leg pain. Similarly, nurses have reported of neck problems (62.4%) and lower back pain (60%) as effects of workload [40]. Our results demonstrated that such physical effects cause discomfort and affect nurses’ capacities to perform.

Related to psychological impact, participants expressed feelings of guilt and anger when faced with situations of high workloads where they could not deliver care appropriately and resulted in negative patient outcomes. Similarly, in a qualitative study which aimed at exploring emotions and feelings in critical care situations nurses reported that they experienced guilt when faced with situations that provided them limited control to help patients due to high workloads [41]. Feelings have been described as signifying moral distress in critical care nurses which can consequently result in emotional exhaustion, affect the ability of critical care nurses to provide proper patient care and perform expected responsibilities and can also increase nurses’ intention to leave the profession [42, 43]. This illustrates that psychological effects posed by high nursing workloads in ICU affects nurse performance and consequently increases the problem of nurse shortages.

Findings from this study show that nurses in the ICU aim to provide comprehensive and quality nursing care to patients; however, a high workload hinders their ability for such quality care provision. These findings have significant implications in strategizing ways of reducing nurse workloads to ensure quality care in the ICU. Policy makers and nurse managers need to increase nurse staffing, prioritise allocation of nurses to ICUs and employ ways of surveying the occurrence of adverse events and errors that will improve patient safety in general.

## Conclusion

The study reveals perceived effects of high nursing workload related to patient care in the ICU at Queen Elizabeth Central Hospital in Malawi. Nurses described various effects of high workload on patients in the unit; compromised care and compromised patient safety. Nurses also described that high nursing workload has negative effects on their wellbeing; physically and psychologically and compromises their capacity to perform which in turn affects patient safety. There is need for collective efforts by government, health care institutions and nurse managers to strategise ways of reducing nurse workloads and monitoring quality of nursing care in ICU in order to improve patient safety.

### Limitations of the study

The main limitation of this study is that the study participants did not include nurse managers who are directly involved in staff organisation who would have highlighted other issues pertaining to issues of high nursing workload in the ICU.

## Data Availability

The datasets used and/or analysed during the current study available from the corresponding author on reasonable request.
